# Barriers to enrollment in pulmonary rehabilitation: medical knowledge analysis

**DOI:** 10.31744/einstein_journal/2021AO6115

**Published:** 2021-10-13

**Authors:** Fernanda Gushken, Luiza Helena Degani-Costa, Thaíz Carolina Pimentel Colognese, Maíra Thomazini Rodrigues, Mayra Zanetti, José Luiz Bonamigo-Filho, Luciana Diniz Nagem Janot de Matos

**Affiliations:** 1 Faculdade Israelita de Ciências da Saúde Albert Einstein Hospital Israelita Albert Einstein São PauloSP Brazil Faculdade Israelita de Ciências da Saúde Albert Einstein, Hospital Israelita Albert Einstein, São Paulo, SP, Brazil.; 2 Hospital Israelita Albert Einstein São PauloSP Brazil Hospital Israelita Albert Einstein, São Paulo, SP, Brazil.

**Keywords:** Rehabilitation, Lung diseases, Patient compliance, Delivery of health care, Referral and consultation, Health knowledge, attitudes, practice

## Abstract

**Objective:**

To assess clinicians’ knowledge about pulmonary rehabilitation, and identify the barriers faced when referring patients with health insurance to pulmonary rehabilitation.

**Methods:**

This was a survey-based cross-sectional study conducted in 2019, at a private reference hospital in São Paulo, Brazil. Eligible participants were physicians registered with the following specialties: internal medicine, geriatrics, cardiology, pulmonology or thoracic surgery.

**Results:**

We collected 72 responses, and 99% of participants recognized chronic obstructive pulmonary disease as a potential indication for pulmonary rehabilitation; less often (75%), they listed interstitial lung disease, bronchiectasis and pulmonary hypertension. Most participants (67%) incorrectly associated pulmonary rehabilitation with lung function improvement, while 28% of cardiologists and 35% of internists/geriatricians failed to recognize benefits on mood disorders. Notably, 18% of participants recommended pulmonary rehabilitation only to patients on supplemental oxygen and 14% prescribed only home physical therapy, patterns more commonly seen among non-respiratory physicians. The three most perceived barriers to referral and adherence were health insurance coverage (79%), transportation to pulmonary rehabilitation center (63%) and lack of social support (29%).

**Conclusion:**

Financial, logistic and social constraints pose challenges to pulmonary rehabilitation enrollment, even for patients with premium healthcare insurance. Moreover, physician knowledge gaps may be an additional barrier to pulmonary rehabilitation referral and adherence. Providing continued medical education, incorporating automatic reminders in electronic medical records, and using telerehabilitation tools may improve pulmonary rehabilitation referral, adherence, and ultimately, patient care.

## INTRODUCTION

Pulmonary rehabilitation (PR) is a complex and individualized intervention that involves a multidisciplinary team of physicians, physical therapists, dietitians and psychologists. It encompasses not only aerobic and resistance training, but also lung expansion and respiratory muscle training, nutritional counseling, basic disease education for the development of self-management strategies, assessment and treatment of mood disorders, as well as smoking cessation programs.^(1)^

The benefits of PR in the treatment of patients with chronic obstructive pulmonary disease (COPD) have been widely studied and confirmed,^(2,3)^ but the success of this therapy is not restricted to this population. Rehabilitation programs also benefit patients with bronchiectasis, pulmonary hypertension, interstitial lung disease and patients pre- and postoperatively after lung resection and lung transplant surgery.^(4,5)^ Proven effects of PR include improvement in quality of life, exercise capacity and anxiety and reduction in depression, frequency of exacerbations and hospitalization rates.^(2,3,6)^ More recently, PR was shown to significantly reduce 1-year mortality following exacerbation of COPD.^(7)^

In this context, it is generally agreed that PR is a highly cost-effective strategy.^(8,9)^ Nevertheless, PR remains largely underutilized worldwide. An American study, for instance, showed that only 3.7% of COPD patients treated by Medicare, in 2012, were in rehabilitation programs,^(10)^ which is consistent with similar international surveys.^(11,12)^ The multiple reasons for this scenario can usually be grouped into one of the following three categories: availability of PR centers, physician awareness, and patient issues impacting on attendance and adherence.

It is estimated that even if all rehabilitation centers in North America, Europe and Australia were operating at full capacity, less than 1.2% of COPD patients could be enrolled.^(13)^ The Brazilian reality is not too encouraging either. In 2017, Brazil had more than 150 cardiopulmonary rehabilitation centers, which seems a lot when compared to other Latin American countries, such as Argentina (32), Colombia (12), Mexico (3) or Uruguay (2).^(13)^ However, given the prevalence of COPD in the Brazilian population aged over 40 years is estimated to be 15%,^(14)^ and that patients with various other chronic lung diseases would also benefit from participation in PR programs, the number of existing centers is still insufficient. Since most of these programs are based at reference hospitals in large cities, they are inaccessible to a considerable proportion of patients and significantly contribute to underutilization of programs.^(10,12,15)^

In principle, many of these barriers should not exist when evaluating patients of high socioeconomic status with access to premium private health insurance. Therefore, we looked hospital admissions and PR enrollment at *Hospital Israelita Albert Einstein* (HIAE), in São Paulo, Brazil, in 2018. While the hospital has been recently acknowledged as one of the leading hospitals in Latin America, only 23 patients per month (on average) underwent PR at its rehabilitation center. During the same period, there were 2,606 admissions related to chronic lung conditions (ICD-10 J40-47, J67, J84), of which 467 listed lung disease as the principal diagnosis.

This data highlighted a clear mismatch between hospital admissions and PR enrollment, suggesting there is room to improve patient care.

## OBJECTIVE

To assess clinicians’ knowledge about pulmonary rehabilitation, and identify the barriers faced when referring patients with health insurance for pulmonary rehabilitation; to investigated if there would be differences regarding knowledge and perceived barriers according to medical specialties.

## METHODS

### Study design and participants

This was a cross-sectional study carried out at HIAE, between May and October 2019. Eligible participants were physicians registered in the study hospital with the following specialties: Internal Medicine, Geriatrics, Cardiology, Pulmonology or Thoracic Surgery. The choice of specialties was based on the fact these physicians are more likely to care for patients requiring PR. For purposes of analysis, respondents were subsequently divided into three groups: Group 1 corresponding to pulmonology and thoracic surgery; Group 2, to cardiology, and Group 3, to geriatrics and internal medicine. Physicians who did not sign the informed consent form were excluded.

A questionnaire was developed by the authors based on results of previous studies^(11,12)^ and their own clinical experience at the organization. It consisted of 11 multiple-choice questions, divided into four categories: knowledge about PR, pattern of referral to PR, barriers to including PR in plans of care; and suggestions to attract more patients to our institutional PR center ( Appendix 1 ). The questionnaire was created using Research Electronic Data Capture (REDCap) platform and sent out to eligible participants by e-mail, while printed versions of the same questionnaire were distributed in scientific meetings of the organization. This study was approved by the Research Ethical Committee of the organization, under protocol 3.182.042, CAAE: 06602819.9.0000.0071.

### Statistical analysis

All survey data were entered into a single database. Missing values were assigned as null. Categorical variables were expressed in percentages and presented in bar graphs. Questionnaire responses were analyzed in the total sample of participants and compared between specialties (Groups 1, 2 and 3). The response frequencies of each of the three groups were computed by χ^2^ test, with Bonferroni adjustments. Because of the exploratory nature of the study, there was no sample size calculation. All analyses were performed using Stata IC 5.2.1.

## RESULTS

We had 80 respondents and excluded eight, who did not sign the Informed Consent Form. Of the remaining 72 participants, 23 belonged to Group 1, 29 to Group 2, and 20 to Group 3. These numbers accounted for 13%, 6.1% and 17.5% of pulmonologists/thoracic surgeons, cardiologists, and geriatricians/internists working at the study hospital, respectively.

**Figure 1:**
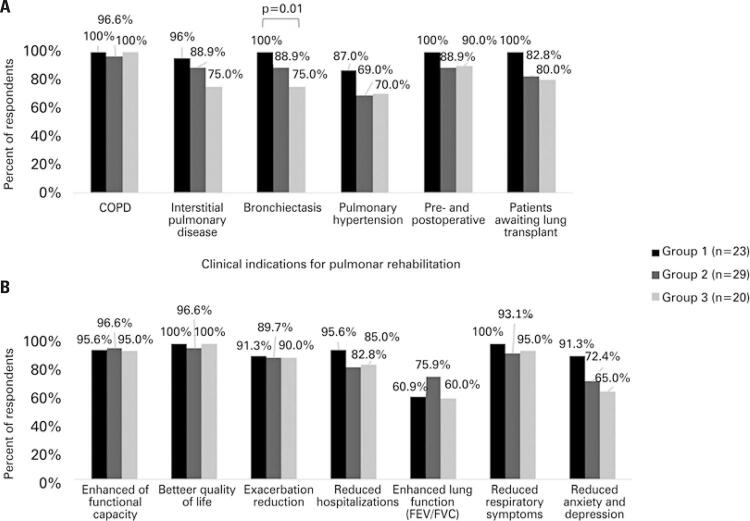
Knowledge about pulmonary rehabilitation, according to medical specialty. A) Knowledge about the indications of pulmonary rehabilitation; B) Knowledge about the benefits of pulmonary rehabilitation

Knowledge about indications and benefits of PR are presented in figure 1. Awareness of PR indication exceeded 69% for all of the six clinical conditions presented, regardless of the participants’ medical specialty. Nevertheless, while COPD was almost unanimously appreciated as a potential indication for PR, interstitial lung disease, bronchiectasis and pulmonary hypertension were less often recognized, especially by Group 3. Additionally, considering the three groups, 99% (71) of all physicians associated PR with COPD patients, whereas only 75% (54) were able to recognize its importance in pulmonary hypertension, although this difference was not statistically significant (Figure 1A). Finally, all (23) participants from Group 1 marked bronchiectasis as a clinical indication to PR, but only 75% (15) from Group 3 did it (p=0.01) (Figure 1A).

Group 1: pulmonology and thoracic surgery; Group 2: cardiology; Group 3: geriatrics and internal medicine.

COPD: chronic obstructive pulmonary disease; FEV: forced expiratory volume; FVC: forced vital capacity.

When it comes to knowledge of the expected benefits of PR, a relatively high percentage of physicians (67% of total respondents, 48) incorrectly associated PR with improvement in lung function (measured by forced expiratory volume in 1 second – FEV_1_ – or forced vital capacity – FVC). There were no statistically significant differences among the groups; however, Group 1 tended to recognize improvement in mood disorders more often than participants in Groups 2 and 3 (91.3% *versus* 72.4% and 65%, respectively) (Figure 1B).

**Figure 2:**
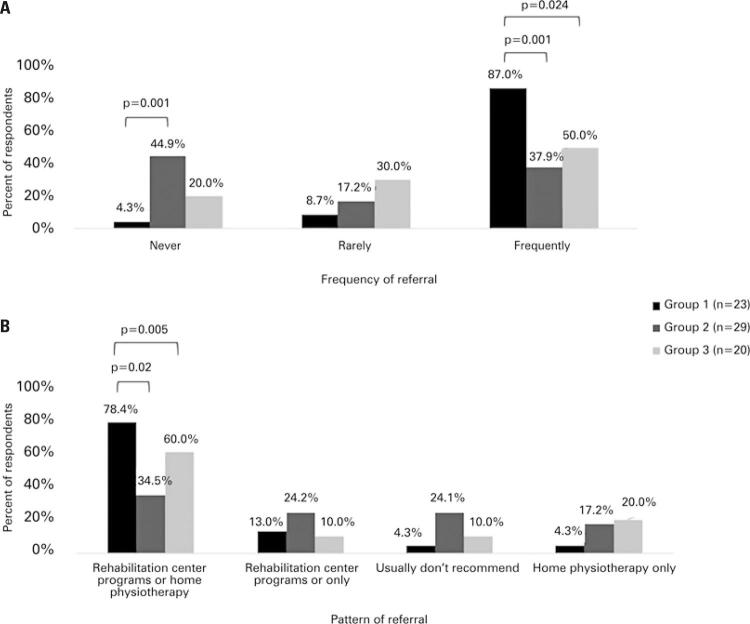
Patterns of referral to pulmonary rehabilitation according to medical specialty. A) Frequency of referral; B) Type of referral

It is noteworthy that 18% (13) of participants suggested rehabilitation only to patients who needed supplemental oxygen, and 14% (10) prescribed only home physical therapy, a patten more commonly seen among cardiologists and internists/geriatricians (Figures 2A and 2B). As expected, pulmonologists and thoracic surgeons referred patients to rehabilitation more often than cardiologists (p=0.001) and geriatricians/internists (p=0.024) (Figure 2A). When they did it, the majority offered both center and home physical therapy, at the discretion of the patient (pulmonologists: 78.4%; cardiologists: 34.5%; p=0.02; internists and geriatricians: 60%; p=0.005 (Figure 2B).

Group 1: pulmonology and thoracic surgery; Group 2: cardiology; Group 3: geriatrics and internal medicine.

**Figure 3:**
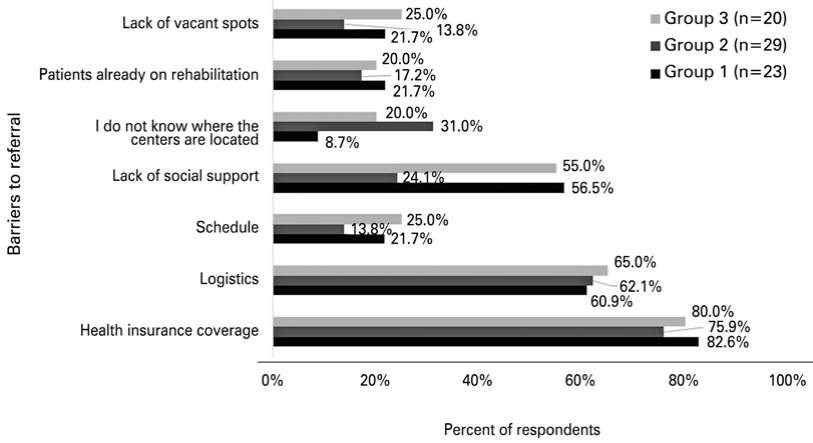
Barriers to referring patients to the pulmonary rehabilitation center at the organization

The majority of participants of the three groups identified issues with health insurance coverage (79.1%; 57) and distance to the PR center (62.5%; 45) as barriers to referring patients to our PR program (Figure 3). Also, lack of social support was mentioned by 38.8% (28) of participants (especially by Groups 1 and 3) (Figure 3), while incompatible timetables due to work responsibilities, lack of vacant spots or patient refusal were less often identified as significant barriers. Interestingly, a significant proportion of cardiologists and internists/geriatricians did not know where to find rehabilitation centers.

Group 1: pulmonology and thoracic surgery; Group 2: cardiology; Group 3: geriatrics and internal medicine.

**Figure 4:**
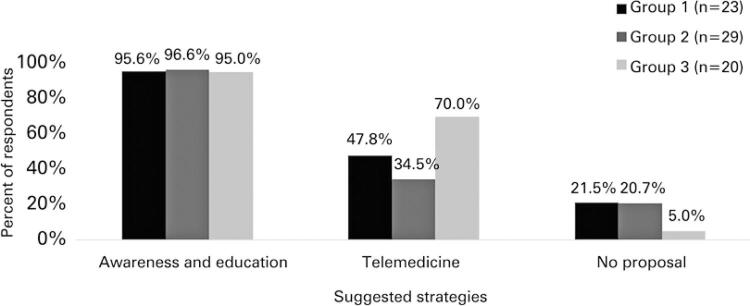
Strategies to increase adherence to pulmonary rehabilitation

When asked about strategies to increase PR referrals, 74% (53) of physicians selected awareness and education, while 46% (33) agreed telemedicine might help. In this regard, Group 3 seemed more open to use telemedicine tools than participants in Group 1 and 2 (Figure 4). In the optional open-ended question about strategies, six participants suggested tackling cost issues broadly, três of them explicitly highlighting the need to negotiate with health insurance providers to expand coverage. Finally, two participants reported that doctors do not receive enough education and information about the benefits of PR, and one participant suggested establishing partnerships with other PR centers.

Group 1: pulmonology and thoracic surgery; Group 2: cardiology; Group 3: geriatrics and internal medicine.

## DISCUSSION

Pulmonary rehabilitation plays a key role in the management of lung diseases. Yet, enrollment in PR programs seems insufficient across the whole socioeconomic spectrum, affecting patients with access to public and private healthcare systems. Having said that, understanding the issues impacting enrollment in each of these contexts is the first step to address the problem correctly. Our study focused on a very specific scenario, seeking to identify barriers to enrollment in PR for patients with access to top private health insurances, in a leading hospital with high-end technology and resources. In our physician survey, the most cited barriers were financial and logistic issues (*e.g.* insufficient health insurance coverage and transportation), as well as lack of social support. Moreover, our results show there is room for improvement in non-respiratory physician knowledge and understanding of PR, especially when it comes to indications in non-COPD patients, and expected benefits apart from improved exercise tolerance.

### Knowledge about recommendations and benefits of pulmonary rehabilitation

While it would be desirable that every respiratory patient could be routinely seen by a pulmonologist, in practice many patients are cared for by geriatricians, internists and cardiologists, both as inpatients and outpatients. Therefore, it is important that these physicians are aware of the indications and benefits of PR. However, our study highlighted a clear knowledge gap between these specialties, when compared to pulmonologists and thoracic surgeons. Although COPD was almost unanimously identified as a potential indication for PR, bronchiectasis, pulmonary fibrosis, and pulmonary hypertension went unnoticed by a relatively high percentage of non-respiratory physicians. Also, roughly 30% of cardiologists, internists and geriatricians simply do not appreciate the benefits of PR with regard to symptoms of anxiety and depression, when in fact breaking the vicious cycle of low functional capacity and social isolation is one of the many advantages of center-based rehabilitation.^(16)^

Furthermore, a high percentage of participants (66%), even respiratory specialists, incorrectly linked PR to lung function improvement, which again suggests many physicians do not have a clear understanding of the components of PR programs, and the mechanisms leading to improved exercise capacity and quality of life. This may explain, at least in part, why almost a fifth of non-respiratory physicians only recommend physical therapy at home to their patients, and never refer them to PR centers.

In that sense, physicians can be considered an important bottleneck in the PR referral process. While educational campaigns (as overwhelmingly suggested by our study participants) might acutely increase awareness, their impact in the long run is less clear, especially for clinicians who do not care for respiratory patients on a daily basis. Therefore, it becomes critical to guarantee that physicians are reminded of assessing eligibility to PR when caring for such patients. Use of clinical support tools within electronic medical records could potentially mitigate this knowledge gap. Best practice alerts, automatically generated from organization guidelines or by algorithms,^(17)^ can aid physicians, suggesting which patients could be referred to PR. If such tools are implemented, it would be essential to balance if the alerts actually help physicians or lead to alert fatigue, cognitive overload and complexity of work.^(18)^

Empowering patients and encouraging them to actively participate in designing their care path is another way of mitigating physician-related barriers to PR referral.^(19)^ When this model is encouraged from the first consultation, the decision-making process will not depend solely on the physician’s perceptions about whether the patient would adhere to a PR program.^(20)^ Once a patient understands the benefits of PR and can actively co-participate in their care planning, the barriers to PR enrollment could be better streamlined. With this approach, the patient, physician and PR team are encouraged to work together to mitigate the barriers, instead of excluding PR as a possibility from the start.^(21)^ Although not addressed in the survey, it is worth analyzing whether hospitalization is effectively used as an opportunity for discussing PR as part of future patient management. After the exacerbation is managed, the patient and support system (family, caregivers) could be more open to discuss a suitable discharge plan, which incorporates enrollment in PR,^(22)^ although the best timing for this approach (during hospital stay, on the day of discharge or on a first follow-up visit) is still very much under debate.

### Perceived barriers to pulmonary rehabilitation referral and adherence

The main barriers to PR referral reported by physicians were financial (79%; 57), logistic (63%; 45) and social (43%; 31). Health insurance coverage was the most cited barrier, reinforcing that even premium plans fail to cover PR, and patients cannot afford to pay for such programs out-of-pocket. Private PR centers should then focus on developing longitudinal cohorts and cost-effectiveness analyses, to help negotiate coverage with insurance companies in a transparent manner, comparing hospitalization and mortality rates of patients who attended PR to those who did not. In many countries, copayments and more flexible packages could be negotiated with insurance companies to increase access to PR programs.^(23)^

Other limiting factors for attending PR were the logistic challenge of attending sessions during work hours and the lack of social support. Commuting to a distant PR center can be especially difficult for a patient who is short of breath and most commonly frail, which means they frequently rely on their relatives, friends or caregivers to take the journey. However, when arranging to attend PR sessions at such distant centers, one has to account not only for the session period, but also for the time in traffic – which, in many cases, could surpass the exercise time itself. While such challenges have been repeatedly reported in the context of public universal healthcare systems, leading to reduced PR uptake and high dropout rates,^(24)^ it seems that physicians perceive them as significant barriers in the private healthcare system as well. This should emphasize the need to investigate the feasibility of expanding the borders of PR centers to allow patients to be treated at home.

Home-based programs have been considered as safe and not inferior to those based on centers, regarding improvement in quality of life related to burden of pulmonary disease (*e.g.* St. George’s Respiratory Questionnaire and Chronic Respiratory Disease Questionnaire), reduction in dyspnea severity (*e.g*. COPD Assessment Test and Modified Medical Research Council Dyspnea Scale), increase in exercise capacity (6 Minute Walk Test) and reduction of anxiety.^(25-27)^ According to the American Thoracic Society (ATS), although home PR alternatives cannot yet be considered a substitute for center based PR, remote solutions become particularly important when the patient is uncapable of meeting the provider face to face.^(28)^ Interestingly, most physicians participating in our survey appeared to be open to incorporating new technologies, such as video-assisted telerehabilitation, to improve patient care.

Many studies have demonstrated feasibility and safety of technology-assisted rehabilitation, using videoconferencing tools or mobile phone applications.^(29)^Unfortunately our survey was carried out in 2019 and until very recently telemedicine was illegal in Brazil. Nonetheless, with the advance of the COVID-19 pandemic, the use of telemedicine was approved in the country, representing a disruptive opportunity to pilot telehealth PR programs. As we see it, technology-supported PR programs could be used not only to replace, but also to add to center-based PR sessions, reducing dropout rates and allowing long-term follow-ups, to prevent patients from going back to baseline exercise capacity after PR is stopped, which is in line with the findings of a recent study performed in Denmark.^(29)^ Moreover, technology-supported PR programs could help create new business models and affect negotiations with health insurance companies.

No single intervention seems to be able to significantly improve PR uptake per se, as pointed out by Barker et al.^(30)^ Rather, a bundle of several process modifications, targeting all potential barriers to enrollment and adherence ought to be put in place in order to achieve the desired results.

### Study limitations

Although the survey was conducted at a single private hospital in Brazil, we believe that our findings shed light into current barriers to PR enrollment for patients with access to private health insurances. Of course, interpretation of our results should take into account the biases inherent to survey studies. For instance, although we managed to demonstrate a significant knowledge gap between respiratory and non-respiratory physicians, the clinical conditions we presented were recognized as potential indications for PR by more than 70% of the physicians, regardless of the participants’ medical specialty. While this could be seen as a good result, it is quite possible that we may have overestimated physicians’ knowledge, by inadvertently selecting those with a greater interest in rehabilitation. Therefore this knowledge gap can be even greater than demonstrated here. Finally, when investigating the barriers to PR enrollment one should ideally seek to understand the point of view of all stakeholders (patients, physicians, PR center directors and health insurance companies), directly assessing their perceptions on the topic. Unfortunately, considering most patients in our organization are cared for by private physicians and not by institutional teams, such a 360^o^ survey would have been hard to carry out. Still, the results of this study are in line with previous international surveys,^(11,12)^ and could provide parameters for future analysis and process redesign.

## CONCLUSION

Physicians identified financial, logistic, and social constraints as significant challenges to enrollment in pulmonary rehabilitation programs, even for patients with access to top healthcare insurance plans. Moreover, our data indicates that non-respiratory physicians have knowledge gaps with regard to the indications of rehabilitation, which may pose as an additional barrier to pulmonary rehabilitation referral and uptake. The results of this study should strengthen the arguments in favor of incorporating a bundle of process modifications and technology-supported solutions, to improve patient care, aiming not only to improve referral to pulmonary rehabilitation, but also to ensure adherence and reduce dropout rates.

## Appendix 1

**Table:** : Structured survey

1. Have you read/ studied/ attended a rehabilitation lecture?
a. Yes.
b. No.
2. For which patient(s) can pulmonary rehabilitation be indicated? (more than one alternative can be marked).
a. COPD.
b. Interstitial lung disease.
c. Bronchiectasis.
d. Pulmonary hypertension.
e. Pre- and postoperative pulmonary resection surgeries.
f. Lung patients awaiting for transplantation.
3. Among the benefits of pulmonary rehabilitation, we can mention the following: (more than one alternative can be marked).
a. Enhanced exercise capacity.
b. Better quality of life.
c. Reduced exacerbations.
d. Reduced hospitalizations.
e. Enhanced pulmonary function (FEV or FVC).
f. Respiratory symptoms reduction.
g. Anxiety and depression reduction.
4. Have you ever referred a patient for pulmonary rehabilitation?
a. Yes.
b. No.
5. How often do you refer patients for pulmonary rehabilitation? Check the alternative that best fits your daily practice.
a. I have never referred any patient.
b. I rarely refer, and these referrals are restricted to very severe and/ or O_2_-dependent patients.
c. I often recommend that my chronic lung disease patients undergo pulmonary rehabilitation, when they maintain flat-walking dyspnea, or have frequent exacerbations regardless of lung function.
6. How many COPD patients do you see, on average, per week?
a. Less than 5.
b. 5 to 10.
c. 10 to 15.
d. More than 15.
7. What is the estimated proportion of your patients who have had or are on pulmonary rehabilitation?
a. 0%.
b. Less than 5%.
c. 5 to 10%.
d. 10 to 20%.
e. 20 to 50%.
f. More than 50%.
8. When you recommend pulmonary rehabilitation for a patient, do you usually:
a. Instruct them to exercise at home with a physical therapist.
b. Recommend inclusion in a rehabilitation center.
c. Offer both options (home physical therapy or rehabilitation center) and let the patient choose the one that suits them best.
d. I do not recommend pulmonary rehabilitation.
9. In your clinical practice, what challenges do you face when referring patients to pulmonary rehabilitation centers? (more than one alternative can be marked).
a. Lack of health insurance coverage.
b. Logistic challenges to take patients to rehabilitation centers due to distance.
c. Patients do not want to participate in programs due to incompatible hours with work schedule.
d. Lack of social support for the patient to attend the sessions (no one available to take them to the center).
e. Patients refuse pulmonary rehabilitation for fear of exercise, or belief they do not need it.
f. Lack of vacant spots for inclusion in rehabilitation programs I know.
g. I do not know how to refer patients to available rehabilitation centers.
h. I do not know where we have pulmonary rehabilitation centers in São Paulo
i. Other: please write down (open answer field).
10. HIAE has a modern rehabilitation center with highly trained professionals. If you have already referred patients to our center, have you found difficult to include your patients into the program?
a. I have never referred any patient to the HIAE rehabilitation center.
b. I have already referred patients and all were included in the program with no difficulty.
c. I have already referred and faced difficulties.
In case you marked option “c”, select what difficulties you faced (more than one alternative can be marked).
a. Cost to the patient.
b. Lack of health insurance coverage.
c. Little availability of schedules/vacant spots in the center.
d. Distance from the patient’s house.
e. Patient found it difficult to schedule by the call center.
f. Other: please specify (open field).
11. Finally, which strategies do you think would be effective in increasing the adherence of pulmonary rehabilitation among your patients?
a. Awareness campaigns with educational materials available in print and/or electronic format.
b. Possibility to perform home rehabilitation via telemedicine.
c. Other: please write down (open field).
