# Supplementary material for: Barriers to enrollment in pulmonary rehabilitation: medical knowledge analysis
Source: Einstein (Sao Paulo). 2021 Oct 13;19:eAO6115. doi: 10.31744/einstein_journal/2021AO6115 (PMC8522705; doi:10.31744/einstein_journal/2021AO6115)
Supplement: Translation

# Translation: Barreiras à inclusão na reabilitação pulmonar: análise do conhecimento médico

**Authors:** Fernanda Gushken, Luiza Helena Degani-Costa, Thaíz Carolina Pimentel Colognese, Maíra Thomazini Rodrigues, Mayra Zanetti, José Luiz Bonamigo-Filho, Luciana Diniz Nagem Janot de Matos

**Affiliations:** 1 Brasil Faculdade Israelita de Ciências da Saúde Albert Einstein, Hospital Israelita Albert Einstein, São Paulo, SP, Brasil.; 2 Brasil Hospital Israelita Albert Einstein, São Paulo, SP, Brasil.

**Keywords:** Reabilitação, Pneumopatias, Cooperação do paciente, Atenção à saúde, Encaminhamento e consulta, Conhecimentos, atitudes e prática em saúde

## Abstract

**Objetivo:**

Avaliar o conhecimento dos médicos sobre reabilitação pulmonar e identificar as barreiras que eles encontram ao encaminhar pacientes com seguro saúde para reabilitação pulmonar.

**Métodos:**

Trata-se de estudo transversal com uso de questionário, realizado em 2019, em um hospital privado de referência em São Paulo. Os participantes elegíveis eram médicos registrados nas seguintes especialidades: clínica médica, geriatria, cardiologia, pneumologia ou cirurgia torácica.

**Resultados:**

Foram coletadas 72 respostas, e 99% dos participantes reconheceram doença pulmonar obstrutiva crônica como possível indicação para reabilitação pulmonar; com menor frequência (75%), listaram doença pulmonar intersticial, bronquiectasia e hipertensão pulmonar. A maioria dos participantes (67%) associou incorretamente a reabilitação pulmonar à melhora da função pulmonar, ao passo que 28% dos cardiologistas e 35% dos clínicos/geriatras deixaram de reconhecer os benefícios nos transtornos de humor. Notavelmente, 18% dos participantes somente recomendaram reabilitação pulmonar para pacientes em uso de oxigênio suplementar, e 14% prescreveram apenas fisioterapia domiciliar, padrão mais comumente visto entre médicos que não são especialistas em transtornos respiratórios. As três barreiras mais percebidas para encaminhamento e adesão foram cobertura de seguro saúde (79%), transporte para centro de reabilitação pulmonar (63%) e falta de apoio social (29%).

**Conclusão:**

Restrições financeiras, logísticas e sociais representam desafios para a inclusão na reabilitação pulmonar, mesmo para pacientes com planos de seguro de saúde da categoria *premium*. Além disso, as lacunas de conhecimento dos médicos podem ser uma barreira adicional para o encaminhamento e a aceitação da reabilitação pulmonar. Educação médica continuada, lembretes automáticos nos prontuários eletrônicos e ferramentas de telerreabilitação podem melhorar o encaminhamento para reabilitação pulmonar, a adesão e, por fim, o atendimento ao paciente.

## INTRODUÇÃO

A reabilitação pulmonar (RP) é uma intervenção complexa e individualizada, que envolve uma equipe multiprofissional de médicos, fisioterapeutas, nutricionistas e psicólogos. Abrange não apenas o treinamento aeróbio e de resistência, mas também a expansão pulmonar e o treinamento muscular respiratório, o aconselhamento nutricional, a educação básica sobre a doença para o desenvolvimento de estratégias de automanejo, a avaliação e tratamento de transtornos do humor e os programas de cessação do tabagismo.^(1)^

Os benefícios da RP no tratamento de pacientes com doença pulmonar obstrutiva crônica (DPOC) foram amplamente estudados e confirmados,^(2,3)^ mas o sucesso dessa terapia não se restringe a essa população. Os programas de reabilitação também beneficiam pacientes com bronquiectasia, hipertensão pulmonar, doença pulmonar intersticial e pacientes no pré- e pós-operatório de ressecção pulmonar e cirurgia de transplante pulmonar.^(4,5)^ Os efeitos comprovados da RP incluem melhora da qualidade de vida, da capacidade de exercício e da ansiedade, além de redução da depressão, da frequência de exacerbações e das taxas de hospitalização.^(2,3,6)^ Recentemente, foi demonstrado que a RP reduz significativamente a mortalidade em 1 ano após exacerbação da DPOC.^(7)^

Nesse contexto, é geralmente aceito que a RP é uma estratégia altamente custo-efetiva.^(8,9)^ No entanto, ela permanece amplamente subutilizada em todo o mundo. Estudo americano, por exemplo, mostrou que apenas 3,7% dos pacientes com DPOC tratados pelo *Medicare*, em 2012, estavam em programas de reabilitação,^(10)^o que é condizente com pesquisas internacionais semelhantes.^(11,12)^ Os vários motivos para esse cenário geralmente podem ser agrupados em uma das três categorias seguintes: disponibilidade de centros de RP, conscientização do médico e problemas do paciente que afetam o atendimento e a adesão.

Estima-se que, mesmo se todos os centros de reabilitação na América do Norte, Europa e Austrália estivessem operando em plena capacidade, menos de 1,2% dos pacientes com DPOC poderiam ser incluídos.^(13)^ A realidade brasileira também não é muito animadora. Em 2017, o Brasil contava com mais de 150 centros de reabilitação cardiopulmonar, o que parece muito quando comparado a outros países latino-americanos, como Argentina (32), Colômbia (12), México (3) ou Uruguai (2).^(13)^ No entanto, levando-se em conta que a prevalência de DPOC na população brasileira com mais de 40 anos é estimada em 15%,^(14)^ e pacientes com várias outras doenças pulmonares crônicas também se beneficiariam da participação em programas de RP, o número de centros existentes ainda é insuficiente. Como a maioria desses programas é implantada em hospitais de referência nas grandes cidades, eles são inacessíveis a uma proporção considerável de pacientes, o que contribui, de modo significativo, para a subutilização dos programas.^(10,12,15)^

Em princípio, muitas dessas barreiras não deveriam existir, ao se avaliarem pacientes de alto nível socioeconômico com acesso a planos de saúde privados da categoria *premium*. Assim, analisaram-se as admissões hospitalares e a inclusão em RP no Hospital Israelita Albert Einstein (HIAE), em São Paulo (SP), em 2018. Embora o hospital tenha sido recentemente reconhecido como um dos principais hospitais da América Latina, apenas 23 pacientes por mês, em média, realizaram RP em seu centro de reabilitação. Durante o mesmo período, ocorreram 2.606 internações relacionadas a doenças pulmonares crônicas (CID-10 J40-47, J67, J84), das quais 467 listaram a doença pulmonar como o diagnóstico principal.

Esses dados destacam uma clara incompatibilidade entre as internações hospitalares e a inclusão na RP, sugerindo que há espaço para melhorar o atendimento ao paciente.

## OBJETIVO

Avaliar o conhecimento dos médicos sobre reabilitação pulmonar e identificar as barreiras que eles encontram ao encaminhar pacientes com seguros de saúde para reabilitação pulmonar. Investigou-se também se existiriam diferenças no que diz respeito ao conhecimento e às barreiras percebidas, de acordo com as especialidades médicas.

## MÉTODOS

### Desenho do estudo e participantes

Trata-se de um estudo transversal realizado no HIAE, entre maio e outubro de 2019. Os participantes elegíveis eram médicos registrados no hospital nas seguintes especialidades: clínica médica, geriatria, cardiologia, pneumologia ou cirurgia torácica. A escolha das especialidades se baseou no fato de esses médicos provavelmente atenderem pacientes com necessidade de RP. Para fins de análise, os respondentes foram posteriormente divididos em três grupos, sendo Grupo 1, correspondente a pneumologia e cirurgia torácica; Grupo 2, à cardiologia e Grupo 3, à geriatria e clínica médica. Os médicos que não assinaram o Termo de Consentimento Livre e Esclarecido foram excluídos.

Um questionário foi elaborado pelos autores com base em resultados de estudos anteriores^(11,12)^ e na própria experiência clínica na instituição. Continha 11 questões de múltipla escolha, divididas em quatro categorias: conhecimento sobre RP, padrão de encaminhamento para RP, barreiras para inclusão da RP nos planos de assistência ao paciente e sugestões para melhorar a captação de pacientes no centro de RP institucional (Apêndice 1). O questionário foi elaborado na plataforma *Research Electronic Data Capture* (REDCap) e enviado aos participantes elegíveis por *e-mail*, sendo que versões impressas do mesmo questionário foram distribuídas em reuniões científicas institucionais. Este estudo foi aprovado pelo Comitê de Ética em Pesquisa da instituição, com protocolo 3.182.042, CAAE: 06602819.9.0000.0071.

### Análise estatística

Todos os dados da pesquisa foram inseridos em um único banco de dados. Os valores ausentes foram atribuídos como nulos. As variáveis categóricas foram expressas em percentagem e apresentadas em gráficos de barras. As respostas do questionário foram analisadas na amostra total de participantes e comparadas entre as especialidades (Grupos 1, 2 e 3). As frequências de resposta de cada um dos três grupos foram calculadas por teste do χ^2^, com ajustes de Bonferroni. Devido à natureza exploratória do estudo, não foi feito cálculo de tamanho amostral. Todas as análises foram realizadas pelo programa Stata IC 5.2.1.

## RESULTADOS

Foram entrevistados 80 participantes, e oito foram excluídos, pois não assinaram o Termo de Consentimento Livre e Informado. Dos 72 participantes restantes, 23 pertenciam ao Grupo 1, 29 ao Grupo 2 e 20 ao Grupo 3. Esses números representaram 13%, 6,1% e 17,5% dos pneumologistas/cirurgiões torácicos, cardiologistas e geriatras/clínicos que trabalhavam no hospital do estudo, respectivamente.

O conhecimento sobre as indicações e os benefícios da RP são apresentados na figura 1. O conhecimento da indicação de RP excedeu 69% para todas as seis condições clínicas apresentadas, independente da especialidade médica dos participantes. No entanto, embora a DPOC tenha sido quase unanimemente reconhecida como possível indicação para RP, doença pulmonar intersticial, bronquiectasia e hipertensão pulmonar foram identificadas com menos frequência, especialmente pelo Grupo 3. Além disso, levando-se em conta os três grupos, 99% (71) de todos os médicos associaram a RP a pacientes com DPOC, ao passo que apenas 75% (54) foram capazes de reconhecer sua importância na hipertensão pulmonar, embora essa diferença não tenha alcançado significância estatística (Figura 1A). Por fim, todos (23) os participantes do Grupo 1 assinalaram bronquiectasia como indicação clínica para RP, mas apenas 75% (15) do Grupo 3 o fizeram (p=0,01) (Figura 1A).

**Figura 1:** Conhecimento sobre reabilitação pulmonar, segundo especialidade médica. A) Conhecimento das indicações de reabilitação pulmonar; B) Conhecimento dos benefícios da reabilitação pulmonar

Grupo 1: pneumologia e cirurgia torácica; Grupo 2: cardiologia; Grupo 3: geriatria e clínica médica.

DPOC: doença pulmonar obstrutiva crônica; VEF: volume expiratório forçado; CVF: capacidade vital forçada.

No tocante ao conhecimento dos benefícios esperados da RP, uma percentagem relativamente alta de médicos (67% do total de entrevistados, 48) associou incorretamente a RP à melhora da função pulmonar (medida pelo volume expiratório forçado em 1 segundo – VEF_1_ – ou capacidade vital forçada – CVF). Não houve diferenças estatisticamente significativas entre os grupos; no entanto, o Grupo 1 tendeu a reconhecer melhora nos transtornos do humor com mais frequência do que os participantes dos Grupos 2 e 3 (91,3% *versus* 72,4% e 65%, respectivamente) (Figura 1B).

Ressalta-se que 18% (13) dos participantes sugeriram reabilitação apenas para pacientes que necessitavam de oxigênio suplementar, e 14% (10) prescreveram apenas fisioterapia domiciliar, padrão mais comumente observado entre cardiologistas e clínicos/geriatras (Figuras 2A e 2B). Como esperado, pneumologistas e cirurgiões torácicos encaminharam pacientes para reabilitação com maior frequência do que os cardiologistas (p=0,001) e geriatras/clínicos (p=0,024) (Figura 2A). Quando o fizeram, a maioria ofereceu fisioterapia tanto no centro quanto em casa, a critério do paciente (pneumologistas: 78,4%; cardiologistas: 34,5%; p=0,02; clínicos e geriatras: 60%; p=0,005) (Figura 2B).

**Figura 2:** Padrões de encaminhamento para reabilitação pulmonar, segundo especialidade médica. A) Frequência de encaminhamento; B) Tipo de encaminhamento

Grupo 1: pneumologia e cirurgia torácica; Grupo 2: cardiologia; Grupo 3: geriatria e clínica médica.

A maioria dos participantes dos três grupos identificou problemas com a cobertura do seguro saúde (79,1%; 57) e a distância para o centro de RP (62,5%; 45) como barreiras para o encaminhamento de pacientes para o programa de RP institucional (Figura 3). Além disso, a falta de apoio social foi mencionada por 38,8% (28) dos participantes (especialmente pelos Grupos 1 e 3) (Figura 3), ao passo que horários incompatíveis devido a responsabilidades de trabalho, falta de vagas ou recusa do paciente foram identificados com menos frequência como barreiras significativas. Curiosamente, uma proporção significativa de cardiologistas e clínicos/geriatras não sabia onde encontrar centros de reabilitação.

**Figura 3:** Barreiras para o encaminhamento de pacientes para o centro de reabilitação pulmonar institucional

Grupo 1: pneumologia e cirurgia torácica; Grupo 2: cardiologia; Grupo 3: geriatria e clínica médica.

Quando questionados sobre estratégias para aumentar os encaminhamentos para RP, 74% (53) dos médicos selecionaram conscientização e educação, ao passo que 46% (33) concordaram que a telemedicina pode ajudar. Nesse sentido, o Grupo 3 parecia mais aberto ao uso de ferramentas de telemedicina do que os participantes dos Grupos 1 e 2 (Figura 4). Na pergunta aberta opcional sobre estratégias, seis participantes sugeriram abordar as questões de custo de forma ampla, três deles destacando explicitamente a necessidade de negociar a expansão da cobertura com as empresas de seguro saúde. Por fim, dois participantes relataram que os médicos não recebem treinamento e informações suficientes sobre os benefícios da RP, e um participante sugeriu o estabelecimento de parcerias com outros centros de RP.

**Figura 4:** Estratégias para aumentar a adesão à reabilitação pulmonar

Grupo 1: pneumologia e cirurgia torácica; Grupo 2: cardiologia; Grupo 3: geriatria e clínica médica.

## DISCUSSÃO

A RP desempenha papel fundamental no tratamento das doenças pulmonares. No entanto, a inclusão em programas de RP parece insuficiente em todo o espectro socioeconômico, afetando pacientes com acesso aos sistemas de saúde públicos e privados. Desse modo, o primeiro passo para resolver o problema corretamente é entender as questões que afetam a inclusão em cada um desses contextos. Este estudo se concentrou em um cenário muito específico, buscando identificar as barreiras para a inclusão em RP de pacientes com acesso aos melhores planos privados de saúde, em um hospital líder, com tecnologia e recursos de ponta. Na pesquisa com médicos, as barreiras mais citadas foram questões financeiras e logísticas (por exemplo: cobertura de seguro saúde e transporte insuficientes), bem como a falta de apoio social. Além disso, os resultados mostraram que há possibilidade para melhorias no conhecimento e na compreensão sobre RP dos médicos não especialistas em transtornos respiratórios, especialmente no que diz respeito às indicações para pacientes sem DPOC e aos benefícios esperados além da melhora da tolerância ao exercício.

### Conhecimento sobre recomendações e benefícios da reabilitação pulmonar

Embora fosse desejável que todo paciente respiratório pudesse ser rotineiramente visto por um pneumologista, na prática, muitos são atendidos por geriatras, clínicos e cardiologistas, tanto em regime de internação quanto em ambulatório. Portanto, é importante que esses médicos estejam cientes das indicações e dos benefícios da RP. No entanto, este estudo evidenciou clara lacuna de conhecimento em meio a essas especialidades, quando comparadas aos pneumologistas e cirurgiões torácicos. Embora a DPOC tenha sido identificada, de modo quase unânime, como uma possível indicação para RP, bronquiectasia, fibrose pulmonar e hipertensão pulmonar passaram despercebidas por uma percentagem relativamente alta de médicos não especialistas em transtornos respiratórios. Além disso, cerca de 30% dos cardiologistas, clínicos e geriatras simplesmente não reconheceram os benefícios da RP em relação aos sintomas de ansiedade e depressão, quando, na verdade, uma das muitas vantagens do centro reabilitação é o rompimento do ciclo vicioso caracterizado por baixa capacidade funcional e isolamento social.^(16)^

Ainda, uma alta percentagem de participantes (66%), mesmo especialistas pneumologistas e cirurgiões torácicos, relacionou incorretamente a RP à melhora da função pulmonar, o que mais, uma vez, sugere que muitos médicos não têm compreensão clara dos componentes dos programas de RP e nem dos mecanismos que levam à melhoria da capacidade de exercício e da qualidade de vida. Isso pode explicar, pelo menos em parte, por que quase um quinto dos médicos não especialistas em doenças respiratórias recomenda somente fisioterapia domiciliar para seus pacientes e nunca os encaminha para centros de RP.

Nesse sentido, o médico pode ser considerado um gargalo importante no processo de encaminhamento para RP. Embora as campanhas educacionais (conforme sugerido pela maioria dos participantes deste estudo) possam aumentar agudamente a conscientização, seu impacto, a longo prazo, é menos claro, em especial para os médicos que não cuidam de pacientes respiratórios todos os dias. Assim, torna-se fundamental garantir que os médicos sejam lembrados da avaliação da elegibilidade para RP ao atender esses pacientes. O uso de ferramentas de suporte clínico nos prontuários eletrônicos de pacientes pode potencialmente mitigar essa lacuna de conhecimento. Os alertas de melhores práticas, gerados automaticamente a partir de diretrizes da instituição ou por algoritmos,^(17)^ podem ajudar os médicos, sugerindo quais pacientes podem ser encaminhados para RP. Se essas ferramentas forem implementadas, é fundamental que haja um equilíbrio para verificar se os alertas realmente auxiliam os médicos ou se levam à fadiga de alertas, à sobrecarga cognitiva e à complexidade do trabalho.^(18)^

Outra forma de atenuar as barreiras relacionadas ao médico para o encaminhamento para RP seria capacitar os pacientes e incentivá-los a participar ativamente do planejamento de seu plano de cuidados.^(19)^ Quando esse modelo é incentivado desde a primeira consulta, o processo de tomada de decisão não depende apenas das percepções do médico sobre a adesão do paciente a um programa de RP.^(20)^ Uma vez que o paciente compreenda os benefícios da RP e possa coparticipar ativamente em seu plano de cuidados, as barreiras para a inclusão na RP podem ser melhor agilizadas. Com essa abordagem, o paciente, o médico e a equipe de RP são incentivados a trabalhar juntos, para mitigar as barreiras, em vez de excluir a possibilidade da RP desde o início.^(21)^ Embora não tenha sido abordado na pesquisa, vale a pena analisar se a hospitalização é efetivamente usada como oportunidade para discutir a RP como parte do futuro manejo do paciente. Depois que a exacerbação for tratada, o paciente, seus familiares e cuidadores podem estar mais abertos para discutir um plano de alta adequado, que incorpore a inclusão na RP,^(22)^ embora ainda esteja em debate qual seria o melhor momento para essa abordagem (durante a internação, no dia da alta ou em uma primeira consulta de acompanhamento).

### Barreiras percebidas no encaminhamento para reabilitação pulmonar e adesão

As principais barreiras ao encaminhamento para RP relatadas pelos médicos foram financeiras (79%; 57), logísticas (63%; 45) e sociais (43%; 31). A cobertura do seguro saúde foi a barreira mais citada, reforçando que mesmo os planos da categoria *premium* não dão cobertura de RP, e os pacientes não podem pagar tais programas por conta própria. Os centros privados de RP devem, então, se concentrar no desenvolvimento de coortes longitudinais e análises de custo-efetividade, para ajudar a negociar a cobertura com as seguradoras de maneira transparente, comparando as taxas de hospitalização e mortalidade de pacientes que participaram da RP com aqueles que não o fizeram. Em muitos países, copagamentos e pacotes mais flexíveis podem ser negociados com as seguradoras, para aumentar o acesso aos programas de RP.^(23)^

Outros fatores limitantes para a realização do RP foram a dificuldade logística de comparecimento às sessões durante o expediente de trabalho e a falta de apoio social. O deslocamento para um centro de RP distante pode ser muito difícil para um paciente que esteja com falta de ar e geralmente frágil, frequentemente dependendo de parentes, amigos ou cuidadores para realizar o trajeto. Porém, ao se organizar para participar de sessões de RP em centros tão distantes, é preciso levar em conta não apenas o período da sessão, mas também o tempo no trânsito — que, em muitos casos, pode ultrapassar o próprio tempo de exercício. Embora essas dificuldades tenham sido relatadas repetidamente no contexto dos sistemas de saúde públicos universais, levando à redução da captação de RP e a altas taxas de abandono,^(24)^ parece que os médicos as percebem como barreiras significativas também no sistema de saúde privado. Isso deve enfatizar a necessidade de investigar a viabilidade de expandir as fronteiras dos centros de RP, para permitir que os pacientes sejam tratados em casa.

Os programas domiciliares foram considerados seguros e não inferiores àqueles realizados em centros, quando se trata de melhoria na qualidade de vida relacionada à carga de doença pulmonar (por exemplo, Questionário Respiratório de St. George e Questionário sobre Doenças Respiratórias Crônicas), à redução na intensidade da dispneia (por exemplo: COPD *Assessment Test* e *Modified Medical Research Council Dyspnoea Scale*), ao aumento da capacidade de exercício (Teste da Caminhada de 6 Minutos) e à redução da ansiedade.^(25-27)^ De acordo com a *American Thoracic Society* (ATS), embora as alternativas de RP em casa ainda não possam ser consideradas um substituto para RP realizada em centros, as soluções remotas tornam-se particularmente importantes quando o paciente não é capaz de estar com o provedor de modo presencial.^(28)^ Curiosamente, a maioria dos médicos participantes desta pesquisa parecia estar receptiva a novas tecnologias, como a telerreabilitação videoassistida, para melhorar o atendimento ao paciente.

Muitos estudos demonstraram a viabilidade e a segurança da reabilitação assistida por tecnologia, usando ferramentas de videoconferência ou aplicativos para telefones celulares.^(29)^ Infelizmente, esta pesquisa foi realizada em 2019, e, até há pouco tempo, a telemedicina era ilegal no Brasil. Entretanto, com o avanço da pandemia COVID-19, o uso da telemedicina foi aprovado no país, representando uma oportunidade inovadora para programas piloto de RP via telessaúde. Entende-se que os programas de RP apoiados por tecnologia podem ser usados não apenas para substituir, mas também para acrescentar às sessões de RP realizadas em centros, reduzindo as taxas de abandono e permitindo acompanhamento de longo prazo, para evitar que os pacientes voltem à capacidade de exercício inicial após a interrupção da RP, o que está de acordo com as descobertas de estudo recente realizado na Dinamarca.^(29)^ Além disso, os programas de RP apoiados por tecnologia podem ajudar a criar novos modelos de negócios e afetar as negociações com seguradoras de saúde.

Nenhuma intervenção isolada parece ser capaz de melhorar significativamente a captação de RP propriamente dita, como apontado por Barker et al.^(30)^ Em vez disso, deveria ser implementado um pacote com várias modificações de processo, visando a todas as possíveis barreiras para inclusão e adesão, de modo a alcançar os resultados desejados.

### Limitações do estudo

Embora a pesquisa tenha sido realizada em um único hospital privado no Brasil, estes resultados lançam luz sobre as barreiras atuais para a inclusão na RP para pacientes com acesso a planos de saúde privados. Obviamente, a interpretação destes resultados deve levar em consideração os vieses inerentes aos estudos de levantamento. Por exemplo, embora se tenha conseguido demonstrar a lacuna de conhecimento significativa entre os médicos especialistas em transtornos respiratórios e os que não o são, as doenças apresentadas foram reconhecidas como possíveis indicações para RP por mais de 70% dos médicos, independente da especialidade médica dos participantes. Embora isso possa ser visto como um bom resultado, é bem possível que se tenha superestimado o conhecimento dos médicos, ao selecionar inadvertidamente aqueles com maior interesse na reabilitação. Portanto, na realidade, essa lacuna de conhecimento pode ser ainda maior do que o demonstrado aqui. Por fim, ao investigar as barreiras para a inclusão em RP, deve-se, idealmente, buscar entender o ponto de vista de todas as partes interessadas (pacientes, médicos, diretores de centros de RP e seguradoras de saúde), em uma avaliação direta de suas percepções sobre o tema. Infelizmente, considerando que a maioria dos pacientes nesta instituição são atendidos por médicos particulares e não por equipes institucionais, essa pesquisa 360^o^ teria sido difícil de realizar. Ainda assim, os resultados deste estudo estão de acordo com pesquisas internacionais^(11,12)^ e podem fornecer parâmetros para futuras análises e um novo desenho de processos.

## CONCLUSÃO

Os médicos identificaram as restrições financeiras, logísticas e sociais como dificuldades significativas para a inclusão em programas de reabilitação pulmonar, mesmo para pacientes com acesso aos melhores planos de saúde. Além disso, estes dados indicam que os médicos não especialistas em doenças respiratórias têm lacunas de conhecimento sobre as indicações de reabilitação, o que pode representar uma barreira adicional ao encaminhamento e à captação da reabilitação pulmonar. Os resultados deste estudo devem fortalecer os argumentos a favor da incorporação de um pacote de modificações de processos e soluções de suporte tecnológico, para melhorar o atendimento ao paciente, visando não só melhorar o encaminhamento para a reabilitação pulmonar, mas também garantir a adesão e reduzir as taxas de abandono.

## Apêndice

**Table:** : Questionário estruturado

1. Você leu/ estudou/ assistiu a uma palestra sobre reabilitação?
a. Sim.
b. Não.
2. Para quais pacientes pode ser indicada a reabilitação pulmonar? (pode marcar mais de uma alternativa).
a. DPOC.
b. Doença pulmonar intersticial.
c. Bronquiectasia.
d. Hipertensão pulmonar.
e. Pré- e pós-operatório de cirurgia de ressecção pulmonar.
f. Pacientes pulmonares aguardando transplante.
3. Dentre os benefícios da reabilitação pulmonar, podemos citar os seguintes: (pode marcar mais de uma alternativa).
a. Maior capacidade para exercícios.
b. Melhor qualidade de vida.
c. Redução de exacerbações.
d. Redução de hospitalizações.
e. Melhora da função pulmonar (VEF ou CVF).
f. Redução de sintomas respiratórios.
g. Redução de ansiedade e depressão.
4. Já encaminhou algum paciente para reabilitação pulmonar?
a. Sim.
b. Não.
5. Com que frequência encaminha pacientes para a reabilitação pulmonar? Assinale a alternativa que melhor se enquadra em sua prática diária.
a. Nunca encaminhei nenhum paciente.
b. Raramente encaminho, e esses encaminhamentos são restritos a pacientes muito graves e/ou dependentes de O_2_.
c. Frequentemente recomendo que meus pacientes com doença pulmonar crônica sejam submetidos à reabilitação pulmonar, quando mantêm dispneia ao caminhar em superfícies planas, ou apresentam exacerbações frequentes, independente da função pulmonar.
6. Quantos pacientes com DPOC você atende, em média, por semana?
a. Menos de 5.
b. 5 a 10.
c. 10 a 15.
d. Mais de 15.
7. Qual é a proporção estimada de seus pacientes que tiveram ou estão em reabilitação pulmonar?
a. 0%.
b. Menos de 5%.
c. 5 a 10%.
d. 10 a 20%.
e. 20 a 50%.
f. Mais de 50%.
8. Ao recomendar a reabilitação pulmonar para um paciente, você geralmente:
a. Instrui a se exercitar em casa com um fisioterapeuta.
b. Recomenda inclusão em um centro de reabilitação.
c. Oferece as duas opções (fisioterapia domiciliar ou centro de reabilitação) e deixa o paciente escolher a que mais lhe convém.
d. Não recomendo reabilitação pulmonar.
9. Em sua prática clínica, quais são as dificuldades que você encontra para encaminhar pacientes para centros de reabilitação pulmonar? (pode marcar mais de uma alternativa).
a. Falta de cobertura de seguro saúde.
b. Dificuldades logísticas de levar os pacientes para os centros de reabilitação devido à distância.
c. Os pacientes não querem participar de programas devido a horários incompatíveis com o expediente de trabalho.
d. Falta de apoio social para o paciente comparecer às sessões (não tem alguém que o leve ao centro).
e. Os pacientes recusam a reabilitação pulmonar por medo de exercícios ou por achar que não precisam.
f. Falta de vagas para inclusão nos programas de reabilitação que conheço.
g. Não sei como encaminhar pacientes para os centros de reabilitação disponíveis.
h. Não sei onde há centros de reabilitação pulmonar em São Paulo.
i. Outro: escreva (campo para resposta aberta).
10. O HIAE possui um moderno centro de reabilitação com profissionais altamente capacitados. Se já encaminhou pacientes para nosso centro, teve dificuldade para incluir seus pacientes no programa?
a. Nunca encaminhei nenhum paciente para o centro de reabilitação do HIAE.
b. Já encaminhei pacientes, e todos foram incluídos no programa sem dificuldade.
c. Já encaminhei e encontrei dificuldades.
Caso tenha marcado a opção “c”, selecione quais dificuldades encontrou. (pode marcar mais de uma alternativa).
a. Custo para o paciente.
b. Falta de cobertura de seguro saúde.
c. Pouca disponibilidade de horários/vagas no centro.
d. Distância da casa do paciente.
e. O paciente teve dificuldade de agendamento pelo *call center*.
f. Outro: especifique (campo aberto).
11. Por fim, quais estratégias acha que seriam efetivas para aumentar a adesão à reabilitação pulmonar de seus pacientes?
a. Campanhas de conscientização com materiais educativos disponíveis em formato impresso e/ou eletrônico.
b. Possibilidade de realizar reabilitação domiciliar via telemedicina.
c. Outro: escreva (campo aberto).
